# Spondyloarthritis - the clinical picture in children

**DOI:** 10.1186/1546-0096-12-S1-I17

**Published:** 2014-09-17

**Authors:** Ruben Burgos Vargas

**Affiliations:** 1 Hospital General de México; 2 Universidad Nacional Autónoma de México, Mexico, Mexico

## 

A group of clinical conditions with the spectrum of adult onset Spondyloarthritis (SpA) characterized at presentation by the inflammatory involvement of the lower limb joints and entheses and in some cases, particularly those with HLA-B27, sacroiliac and spinal joint involvement. Enthesitis Related Arthritis (ERA) and Psoriatic Arthritis (PsA), both categories of Juvenile Idiopathic Arthritis correspond to juvenile-onset SpA at some extentWith the advent of new classification criteria for axial SpA (axSpA) and peripheral SpA (pSpA) proposed by the Assessment of SpondyloArthritis internationalSociety (ASAS) to identify patients with ankylosing spondylitis in the earliest pre-radiographic stage of the disease to treat them with Tumor Necrosis Factor (TNF) alpha blockers and halter disease progression, there is a tendency to search for early sacroiliac involvement with Magnetic Resonance Imaging (MRI) and peripheral disease with Ultrasonographic (US) studies in childrenThere is clearly a risk in trying to resemble the objective and procedures developed for the adult patients in the pediatric population. In the first place, the most frequent and often severe inflammatory and structural changes in children and adolescents with SpA occur at peripheral sites five to 10 years before the onset of axial disease. Peripheral involvement might be so severe that TNF blockers may be indicated years before sacroiliitis and spondylitis. It is therefore obvious that searching for axial disease with MRI may be useless.Both anatomy and biomechanics of peripheral sites and axial skeleton change throughout childhood and adolescence. In consequence, it is important to understand basic aspects of the sacroiliac and spinal joints before developing plans to search such sites to detect inflammation and prescribe TNF blockers.

## Disclosure of interest

None declared.

